# Supplementary material for: Development of MPFC function mediates shifts in self-protective behavior provoked by social feedback
Source: Nat Commun. 2018 Aug 6;9:3086. doi: 10.1038/s41467-018-05553-2 (PMC6079030; doi:10.1038/s41467-018-05553-2)
Supplement: Supplementary file 1 — Supplementary Information [file 41467_2018_5553_MOESM1_ESM.pdf]

Supplementary Information

Development of MPFC function mediates shifts in self-protective behavior provoked by social feedback

Yoon et al.

Supplementary Note 1. Post-experimental questionnaires designed to assess participant-specific task experience.

For exploratory purpose, we measured each participant's interest, effort, self-evaluation, and expected other-evaluation immediately following the Artwork creation task. The exact questions include: 1) "How interesting was this activity? [0 (not interesting at all) - 10 (very interesting)]," 2) "How much effort did you put on this activity? [0 (No effort at all) - 10 (Very much effort)]," 3) "How creative do you think your artwork is? [0 (Not creative at all) - 100 (Very creative)]," 4) "How many people (out of 100 people) do you expect would evaluate your artwork as creative? [0, 5, 10, 15 … 100 people]."

Supplementary Note 2. Analysis testing the sensitivity to objective creativity (OC) in the Artwork evaluation task among the minor participants.

To ensure that the minor raters ($N = 45$) were not insensitive to the creativity of artworks determined by the independent adult raters, we ran a repeated measures ANOVA with an independent variable of 5 levels of OC determined by the independent adult sample and a dependent variable of proportion of favorable evaluation. The result showed a robust effect of OC on the evaluation of the artworks ($F(4, 41) = 41.905$, $p < 0.001$), suggesting that the minor participants were sensitive to the creativity of artworks, similarly to the independent adult group.

Supplementary Note 3. Post-experimental questionnaires indirectly probing suspicion about the purpose of the study.

To assess whether the participants were aware of the purpose of the study or not, we asked the participants to answer the following post-experimental questions: 1) "Write down your thoughts and feelings during the task freely." 2) "How did you feel when the partner evaluated your artwork not creative? [0 (I didn't feel bad at all) – 5 (I felt very bad)], and write down the reason of your answer as specifically as possible (open question)." 3) "How did you feel when the partner evaluated your artwork creative? ([0 (I didn't feel good at all) – 5 (I felt very good)], and write down the reason for your answer as specifically as possible (open question)." We expected that participants suspicious of the cover story would report no emotional response to the feedbacks and provide their suspicion as reasons for such responses in the following open questions. Indeed, no participant reported any doubt in the open questions.

Supplementary Note 4. Measurement of task-related personality trait variables.

We measured various personality trait variables that are potentially related to the behavioral outcomes in the Artwork evaluation task. First, we asked how important it is for them to be creative in general [1 (Not important at all) - 6 (Very important)], which would indicate individual differences in the perceived significance of the trait of creativity. Second, participants reported approval need and trait self-esteem using a 20-item of the Revised Martin-Narsen Approval Motivation Scale (RMLAM)[1] and a 10-item of the Rosenberg Self-Esteem Scale[2].

Supplementary Note 5. Details in task structure of the independent adult-only study distinguished from those of the main developmental study.

1) The Reciprocal Artwork Evaluation Task included a total of 99 trials (including those artworks with large between-rater variability): 35 positive feedback trials, 35 negative feedback trials, and 29 neutral feedback trials. 2) The presentation order of all the condition trials was fully randomized such that every participant experienced a unique sequence of the condition trials, which were determined by specific combinations of feedback valence types and OC levels.

Supplementary Note 6. The examples of the younger participants' answers to the opened-questions during the debriefing asking emotional experience when receiving negative feedback from partners.

"I felt bad because I believe people did not see my artwork in detail.", "I felt bad because I received negative evaluations despite the large effort", "I felt bad as it is usual to feel bad when receiving negative words about one's own work", "I couldn't feel good enough when receiving negative evaluation.", "It was frustrated that some people did not acknowledge my effort", "I felt very bad and irritated", "Although I knew that I didn't do very well, I did not feel good with negative evaluation.", "I felt somewhat bad because some people gave me negative evaluation even though they also did not do well", "I thought some people might not have seen my artwork in detail and gave me negative evaluation, because I drew the artwork very elaborately.", "I felt good because many people gave me good evaluation, but I felt somewhat bad because some people gave me negative evaluation", "I also thought my artwork was not that creative. As it was my artwork, however, I felt somewhat bad when receiving negative evaluation."

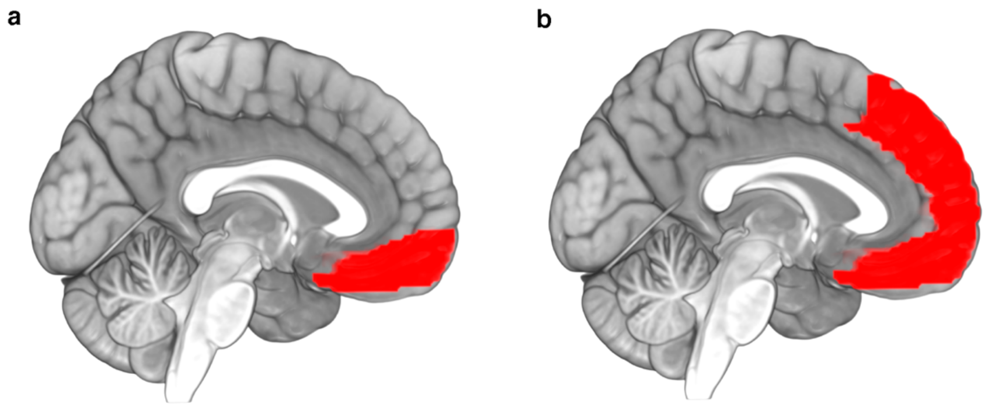

Supplementary Figure 1. Anatomically defined masks used for region-of-interest analysis. (a) Anatomically defined VMPFC used for finding neural activation predicting cFB influence **(b)** Anatomically defined MPFC used for finding neural activation predicting accFB influence and feedback integration by Reinforcement Learning.

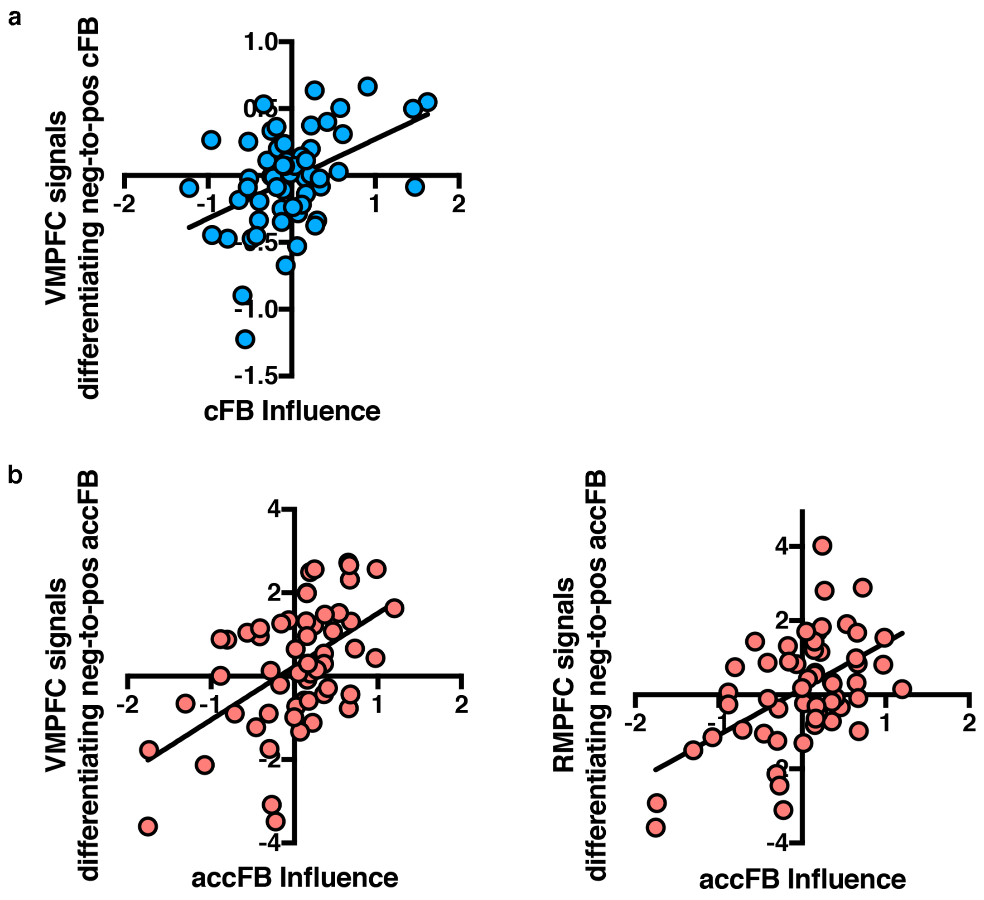

Supplementary Figure 2. Relationships between behavioral and neural indices of self-protective biases. (a) Scatter plots showing the relationship between the behavioral cFB indices and the extent to which signals of VMPFC negatively correlated with the cFB value with the influence of accFB and OC controlled for (one outlier in the VMPFC activity was excluded from this plot). **(b)** Scatter plots showing the relationships between the behavioral accFB indices and the extent to which VMPFC activity (left) and RMPFC activity (right) negatively correlated with the accFB value with the influence cFB and OC controlled for (one outlier in the VMPFC and RMPFC activity was excluded from each plot).

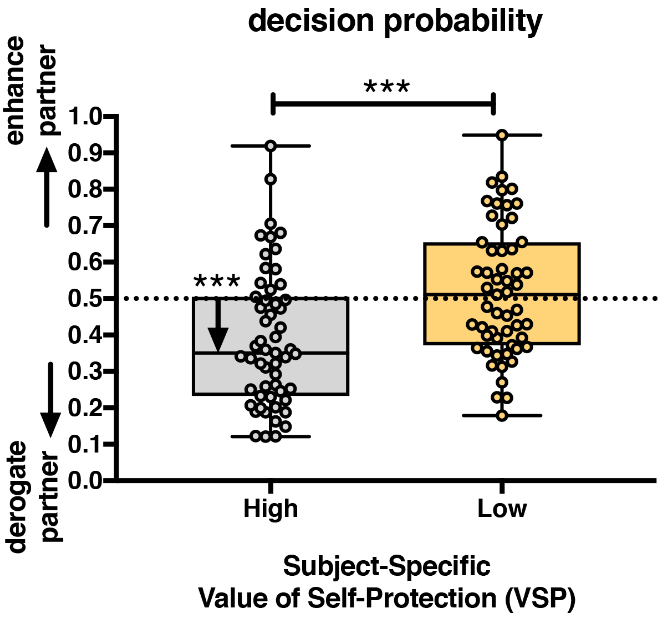

Supplementary Figure 3. Significance of self-protective motivation affecting partner-evaluation bias due to social feedback(s). The impact of Value of Self-Protection (VSP) estimated by the RL model on partner-evaluation was significant only for high ($z = -3.854$, $p < 0.001$) but not for low ($z = 0.462$, $p = 0.645$) VSP trials, and there was a significant difference between the two valence conditions ($z = 5.446$, $p < 0.001$). Dotted line indicates the chance level ($p = 0.5$), the arrow located above the high VSP condition indicates significant partner-derogation (***$p < 0.001$). The lower and upper lines of the box indicate the 25th and 75th percentiles of the distribution, and the middle line represents the median value.

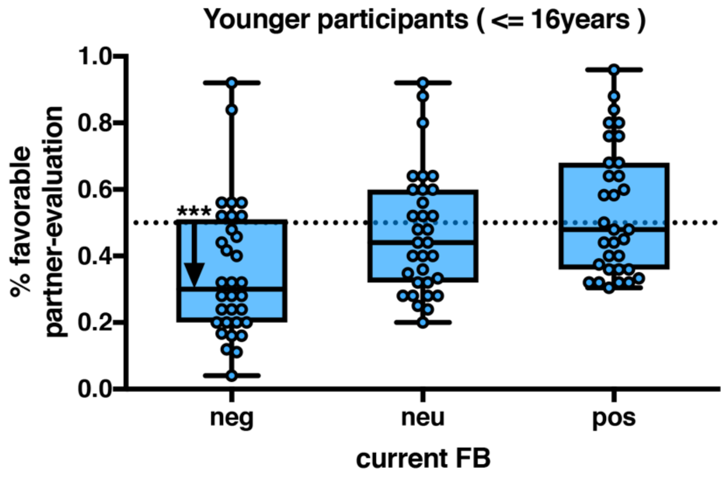

Supplementary Figure 4. The mean proportion of favorable partner-evaluation of the younger participants (<= 16years) following different feedback valence (i.e., negative, neutral, and positive feedback). These data provide evidence that the younger participants' immediate feedback effect reflects self-protective motivation rather than simple copying of their partner's feedbacks, because the proportion of matching feedbacks was significantly above the chance level only following negative (z = -3.294, p < 0.001), but not positive (z = 0.765, p = 0.444), feedbacks. The dotted line indicates the chance level of favorable evaluation (p = 0.5). The arrow indicates increase in negative partner-evaluation significantly above the chance level (***p < 0.001). The lower and upper lines of the box indicate the 25th and 75th percentiles of the distribution, and the middle line represents the median value.

Supplementary Table 1. Age and gender information of participants.

Age	# of each Age (# of each gender)
10	2 (M: 2, F: 0)
11	6 (M: 3, F: 3)
12	3 (M: 2, F: 1)
13	6 (M: 2, F: 4)
14	8 (M: 4, F: 4)
15	2 (M: 0, F: 2)
16	5 (M: 2, F: 3)
17	5 (M: 2, F: 3)
18	8 (M: 5, F: 3)
20	1 (M: 1, F: 0)
21	1 (M: 0, F: 1)
22	3 (M: 2, F: 1)
23	3 (M: 2, F: 1)
24	2 (M: 1, F: 1)
25	3 (M: 1, F: 2)

Supplementary Table 2. List of brain structures identified at the significance level of $p < 0.05$ (Small Volume FWE-corrected or Whole-Brain Gray Matter FWE-corrected). Regions were labelled according to the Automatic Anatomical Labeling version 2 (AAL2) using bspmview (http://www.bobspunt.com/software/bspmview/) and additionally labelled according to the Brodmann atlas using MRIcron (http://people.cas.sc.edu/rorden/mricron/index.html). Different extents of clusters for SVC and Whole-Brain FWE results were distinguished by slash.

Region Label		Extent	t-value	x	y	z	Correction Method (Desired Cluster Extent)
AAL2	BA						
**Regions differentiating neg-to-pos cFB at the feedback receipt event which predicted cFB influence**							
Rectus_R	11	23	3.749	10	24	-18	SVC (17.8)
**Regions differentiating neg-to-pos accFB at the partner-evaluation event which predicted accFB influence**							
Frontal_Sup_Medial_L	10	194 / 197	4.157	-4	62	16	SVC (18) / Whole-Brain (193.7)
Frontal_Med_Orb_L	11	23	3.529	-6	36	-14	SVC (18)
**Regions coding prediction error signals which were negatively associated with learning rate**							
Frontal_Sup_Medial_L	10	539 / 618	4.883	-6	62	10	SVC (22.7) / Whole-Brain (109.9)
Frontal_Med_Orb_L	11	539 / 618	4.743	-4	36	-10	SVC (22.7) / Whole-Brain (109.9)
Rectus_R	11	57	4.211	10	50	-18	SVC (22.7)
Precuneus_R	23/26/30	144	4.247	6	-54	26	Whole-Brain (109.9)

Supplementary Table 3. Descriptive statistics (Mean and Standard Deviation) of proportion of negative evaluation throughout the task, after negative/neutral/positive cFB, after negative/neutral/positive accFB of total sample, younger youth and older youth.

		Total sample (*N*=58)	Younger youth (Age<=16, *N*=32)	Older youth (Age>16, *N*=26)
Proportion of Negative Evaluation	All trials	0.54 (0.17)	0.55 (0.17)	0.53 (0.18)
	Following negative cFB	0.6 (0.21)	0.65 (0.20)	0.54 (0.22)
	Following neutral cFB	0.53 (0.18)	0.53 (0.18)	0.53 (0.17)
	Following positive cFB	0.49 (0.2)	0.46 (0.19)	0.52 (0.21)
	Following negative accFB	0.54 (0.19)	0.53 (0.19)	0.57 (0.19)
	Following neutral accFB	0.55 (0.18)	0.55 (0.17)	0.56 (0.18)
	Following positive accFB	0.53 (0.18)	0.56 (0.18)	0.49 (0.19)

Supplementary Table 4. The relationships between the behavioral indices from the main task (i.e., cFB and accFB effect) and all the other measurements obtained for exploratory purpose. The significance level was Bonferroni-corrected for multiple tests of correlation and no significant association was found with any behavioral measure (the numbers indicate correlation coefficients). For specific contents of the subjective reports on task experiences and task-related personality trait, see Supplementary Note 1 and Note 4.

<table>
<tr><th colspan="2">Exploratory measurements</th><th>cFB effect</th><th>accFB effect</th></tr>
<tr><td>Additional index for developmental progress</td><td>Pubertal Developmental Scale (PDS)</td><td>-0.229</td><td>0.237</td></tr>
<tr><td rowspan="4">Task experience</td><td>Interest</td><td>0.014</td><td>-0.206</td></tr>
<tr><td>Effort</td><td>0.156</td><td>-0.179</td></tr>
<tr><td>Self-Evaluation</td><td>0.132</td><td>-0.162</td></tr>
<tr><td>Expectation of Other-Evaluation</td><td>0.044</td><td>0.029</td></tr>
<tr><td rowspan="3">Task-related personality trait variables</td><td>Perceived significance of creativity</td><td>0.143</td><td>-0.087</td></tr>
<tr><td>Need for Approval</td><td>-0.105</td><td>0.021</td></tr>
<tr><td>Self-Esteem</td><td>0.139</td><td>-0.355</td></tr>
</table>

Supplementary References

1 Martin, H. J. A revised measure of approval motivation and its relationship to social desirability. *J. Pers. Assess.* **48**, 508-519 (1984).

2 Rosenberg, M., Schooler, C. & Schoenbach, C. Self-esteem and adolescent problems: Modeling reciprocal effects. *Am. Sociol. Rev.* **54**, 1004-1018 (1989).
